# Interactions between Conscious and Subconscious Signals: Selective Attention under Feature-Based Competition Increases Neural Selectivity during Brain Adaptation

**DOI:** 10.1523/JNEUROSCI.3052-18.2019

**Published:** 2019-07-10

**Authors:** Yukiko Kikuchi, Jennifer Ip, Gaëtan Lagier, James C. Mossom, Sukhbinder Kumar, Christopher I. Petkov, Nick E. Barraclough, Quoc C. Vuong

**Affiliations:** ^1^Institute of Neuroscience, Henry Wellcome Building, Newcastle University, Framlington Place, Newcastle upon Tyne NE2 4HH, United Kingdom, and; ^2^Department of Psychology, University of York, Heslington, York YO10 5DD, United Kingdom

**Keywords:** adaptation, attention, audition, automatic, prediction, vision

## Abstract

Efficient perception in natural environments depends on neural interactions between voluntary processes within cognitive control, such as attention, and those that are automatic and subconscious, such as brain adaptation to predictable input (also called repetition suppression). Although both attention and adaptation have been studied separately and there is considerable knowledge of the neurobiology involved in each of these processes, how attention interacts with adaptation remains equivocal. We examined how attention interacts with visual and auditory adaptation by measuring neuroimaging effects consistent with changes in either neural gain or selectivity. Male and female human participants were scanned with functional magnetic resonance imaging (fMRI) first while they discriminated repetition of morphed faces or voices and either directed their attention to stimulus identity or spatial location. Attention to face or voice identity, while ignoring stimulus location, solely increased the gain of respectively face- or voice-sensitive cortex. The results were strikingly different in an experiment when participants attended to voice identity versus stimulus loudness. In this case, attention to voice while ignoring sound loudness increased neural selectivity. The combined results show that how attention affects adaptation depends on the level of feature-based competition, reconciling prior conflicting observations. The findings are theoretically important and are discussed in relation to neurobiological interactions between attention and different types of predictive signals.

**SIGNIFICANCE STATEMENT** Adaptation to repeated environmental events is ubiquitous in the animal brain, an automatic typically subconscious, predictive signal. Cognitive influences, such as by attention, powerfully affect sensory processing and can overcome brain adaptation. However, how neural interactions occur between adaptation and attention remains controversial. We conducted fMRI experiments regulating the focus of attention during adaptation to repeated stimuli with perceptually balanced stimulus expectancy. We observed an interaction between attention and adaptation consistent with increased neural selectivity, but only under conditions of feature-based competition, challenging the notion that attention interacts with brain adaptation by only affecting response gain. This demonstrates that attention retains its full complement of mechanistic influences on sensory cortex even as it interacts with more automatic or subconscious predictive processes.

## Introduction

Operating successfully in natural environments requires adaptable processing of goal-relevant and predictable sensory evidence. Attention and adaptation are important in these regards, but how these neurobiological processes interact remains controversial.

Adaptation, also called “repetition suppression” or “stimulus-specific adaptation” (SSA), is prevalent in the animal brain, characterized by neurons reducing their activity to stimulus repetition or redundant repeating events (Malmierca et al., 2015). Although the functional role of adaptation is debated, ranging from familiarity-based memory to neural calibration to environmental statistics, the consensus view is of an automatic form of prediction not requiring consciousness (Barlow and Foldiak, 1989; Clifford et al., 2000; Bekinschtein et al., 2009; Bastos et al., 2012). In humans, fMRI is often used to investigate adaptation [fMR-adaptation (fMR-A); Grill-Spector et al., 1999; Barron et al., 2013; Larsson and Harrison, 2015], exploiting paradigms that reduce hemodynamic responses to stimulus repetition compared with stronger responses to repetition of stimuli with different features. The dominant theoretical explanations for fMR-A effects are “fatigue” or “sharpening” models (Grill-Spector et al., 2006). The fatigue model describes systematic response reduction throughout the stimulus driven neuronal population (reduction in gain). In comparison, the sharpening model describes changes in selectivity or tuning, with some neuronal responses being enhanced while others reduced. By contrast, voluntary goal-directed attention can affect neural gain or selectivity under conditions that are well described; resulting in more selective responses when spatially overlapping features compete for attention and neural processes (Desimone, 1998; Maunsell and Treue, 2006; Okamoto et al., 2007; Reynolds and Heeger, 2009).

However, whether attentional interactions during adaptation result in gain or selectivity changes remains equivocal. There is evidence that neural adaptation can be modified by top-down feedback via attention, expectation, and learning (Murray and Wojciulik, 2004; Summerfield and de Lange, 2014; Barron et al., 2016). Most studies show that attention can affect fMR-A primarily by nonselectively boosting the gain of sensory signals during adaptation (for review, see Summerfield and de Lange, 2014), in essence counteracting the gain reduction induced by adaptation (Eger et al., 2004; Altmann et al., 2008). However, some authors have found that attention increases neural selectivity during fMR-A (Murray and Wojciulik, 2004; Weiner et al., 2010). Furthermore, when assessing how adaptation interacts with attention in the brain, it is crucial to control for other predictive processes, such as the participant's expectation of whether a repetition will occur, which could also increase neural selectivity (Spratling, 2008; Kok et al., 2012b; Summerfield and de Lange, 2014). Thus, a timely outstanding question is as follows: how do attention and adaptation interact at a neural level? The prior conflicting results may indicate that cognition differentially counteracts fMR-A under conditions that require explication. In particular, a critical outstanding issue is whether the modulatory effects of attention on adaptation are dependent upon whether attended stimulus features are in competition with each other or not (Altmann et al., 2008).

To address this issue, we first manipulated the focus of attention in two sensory modalities (visual or auditory) while participants were scanned with fMRI as they either attended to face or voice identity or changes in stimulus spatial location (Experiment 1: nonspatial vs spatial selective attention). During the attention task, we systematically manipulated fMR-A while balancing for the expectation of a stimulus change or no-change for each individual. These experiments in either modality only resulted in attentional gain increases during adaptation in high-level sensory cortex, possibly because attentional processes to spatial versus nonspatial features were under minimal competition. Therefore, in Experiment 2 we manipulated attention to voice identity versus sound loudness (both nonspatial features intrinsic to the complex stimulus), which, unlike the observations from Experiment 1, resulted in increased neural selectivity during adaptation. Altogether, the results indicate that top-down attention can differentially counteract neural adaptation effects via changes in gain or neural selectivity contingent on the level of feature-based competition.

## Materials and Methods

### 

#### Experiment 1

##### Participants.

Twelve volunteers (7 females, age 22 ± 1.4 SD; 5 males age 21 ± 2.0 SD) participated in Experiment 1, with each conducting the experiment in both the auditory or visual modality across two separate 1 h fMRI scanning sessions (24 total scanning sessions). Modality order was counterbalanced across participants. Participants provided informed consent, had normal or corrected-to-normal vision, and reported normal hearing. Ethical approval for the study was awarded by the Newcastle University Ethical Review Committee, and all research was performed in accordance with the ethical standards laid down in the 1990 Declaration of Helsinki.

##### Stimuli.

**Figure 1.**
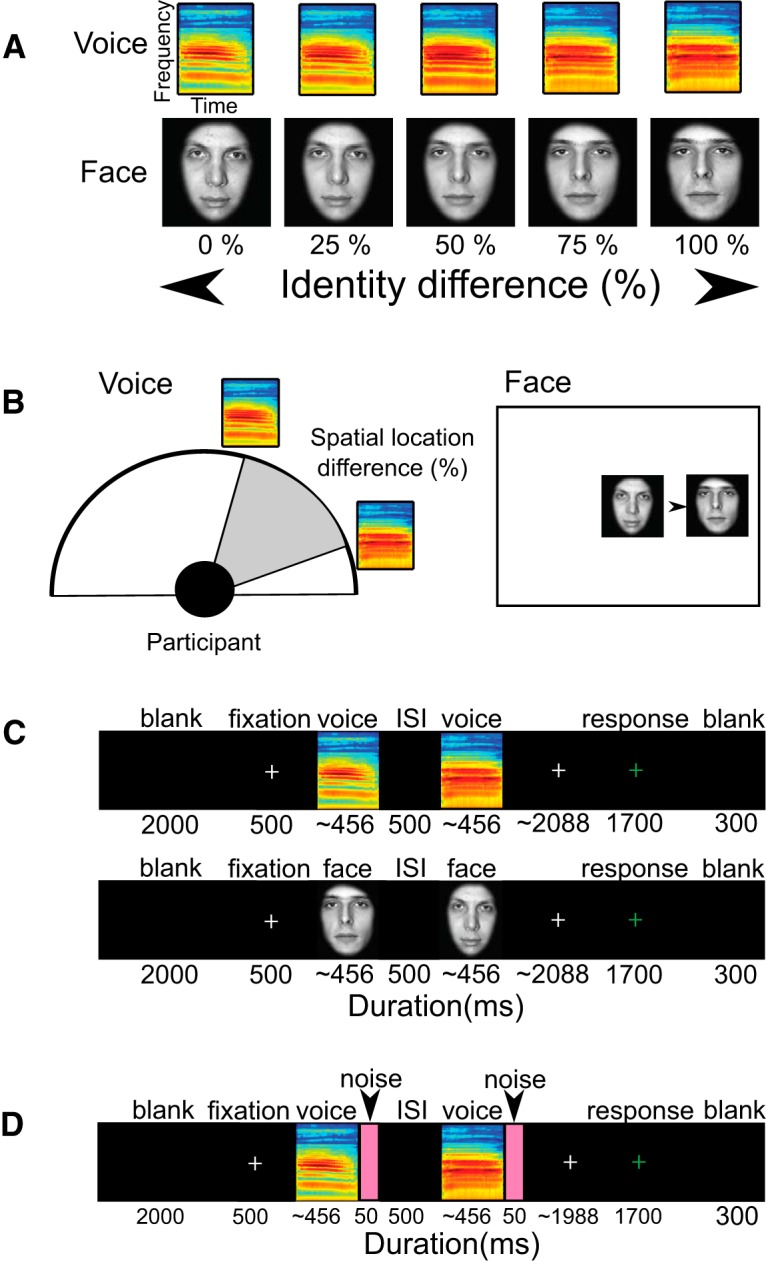
Attention tasks and adaptation conditions for the two experiments. ***A***, Identity change in Experiment 1. Seven identity distances (0–75%) were used for behavioral experiments and three perceptual distance were selected for fMRI experiments. ***B***, Spatial change in Experiment 1. Seven spatial locations on the display monitor for faces or in the virtual acoustic space for voices were selected for behavioral experiment and three perceptual spatial distances were selected for fMRI experiments. ***C***, Time course of Experiment 1. ***D***, Time course of Experiment 2.

Figure 1, *A* and *B*, illustrates the face and voice stimuli and how identity and spatial position were systematically manipulated. The auditory stimuli consisted of emotionally neutral “Ah” utterances from six female and six male speakers recorded in a sound-attenuated room using an Edirol R-09HR voice recorder (Roland), sampled at 44.1 kHz and 32-bit resolution. Each female voice was arbitrarily paired with a male voice to generate six voice pairs that differed maximally. We used TANDEM-STRAIGHT (Kawahara et al., 2008; Kawahara and Morise, 2011) to morph between voices in each pair to create six voice identity continua. In brief, spectral envelope, fundamental frequency, and aperiodicity parameters were extracted from the voice sounds. Each parameter was aligned to match between different voices and manipulated parametrically between the two voices of each pair from 0% (male voice) to 100% (female voice) in 5% steps (Fig. 1*A*), giving a total of 21 voice stimuli per continuum and 126 stimuli in total (6 voice pairs × 21 voice stimuli). We next used REAPER software (http://www.reaper.fm/) to spatially manipulate each voice stimulus so that it was perceived to originate from 40 virtual positions along the azimuth from left (−90°) to right (+90°) in 4° steps (with constant elevation of 0°). Thus, in total, there were 5040 auditory stimuli (40 spatial position × 126 voice stimuli). This manipulation allowed us to present voice pairs from different spatial positions in virtual acoustic space (Fig. 1*B*). The average duration of each stimulus was 456 ± 120 ms (mean ± SD). Finally, the voice stimuli were down sampled to 16 bits, gated with a 20 ms cosine ramp (to shape sound onset and offset), and root mean square normalized following direct-current offset correction.

The visual stimuli consisted of grayscale images of six female and six male faces (Goffaux et al., 2005). Faces had emotionally neutral expressions and were masked to show only internal facial features (i.e., no hair and ears). Each female face was arbitrarily paired with a male face to create six face pairs. We used FantaMorph software v5.0 (Abrosoft) to morph between face pairs to create six face identity continua. In brief, we first manually marked 93 corresponding facial features for each of the 12 faces (e.g., corner of eyes, tip of the nose, etc.) to establish a meaningful correspondence across faces regardless of size and facial shape. Next, we took a weighted average between the paired faces from 0% (male face) to 100% (female face) in 5% steps at each facial feature point (Fig. 1*A*). Thus, there were 21 face images per face pair and 126 face images in total. Finally, we used the SHINE toolbox (Willenbockel et al., 2010) to equalize the luminance across the 126 face images. As the duration of each voice varied, to ensure that the duration of presentation of the faces were equivalent, each of the 126 faces was arbitrarily paired with one of the voices and presented for that duration. Face stimuli subtended ∼8.4° (w) × 7.1° (h) visual angle. To manipulate spatial position, each face image was presented vertically centered in the left or right half of the screen with a horizontal spatial offset relative to each other (Fig. 1*B*).

##### Apparatus.

Participants were trained in a mock scanner environment (Psychology Software Tools) at Newcastle University and tested in a 3-Tesla scanner at the Newcastle Magnetic Resonance Centre (Philips). For the mock scanner, auditory stimuli were presented at ∼75 dB SPL (calibrated with an XL2 sound level meter, NTI Audio) through Sennheiser 380 headphones using an Edirol external sound card (Roland). Visual stimuli were presented on a 19-inch flat-panel monitor at the head of the scanner at 1280 × 1024 pixel resolution. Participants viewed the monitor through a mirror positioned within the mock scanner head coil. They responded using a keypad while in the mock scanner.

At the 3-Tesla scanner, auditory stimuli were presented using an MR-compatible audio system and delivered with electrostatic transducer headphones (NordicNeuroLab). Participants wore earplugs to further shield against scanner noise. Visual stimuli were back-projected onto a screen at the foot of the scanner using a Canon XEED LCD projector (1280 × 1024 pixels). Participants viewed the projection through an angled mirror attached to the head coil ∼10 cm above their eyes. Head motion was restricted by placing foam pads between the head and the head coil. Participants responded via an MR-compatible response pad. The experiments were programmed in MATLAB (MathWorks) with Psychtoolbox (Brainard, 1997; Pelli, 1997).

##### Task and calibration procedure to balance stimulus change expectancy.

In Experiment 1, participants discriminated the identity or spatial position of sequentially presented face or voice pairs. They were instructed to attend to one of these stimulus features and judge whether the attended feature was the same or different, while ignoring changes to the unattended feature. Differences in both identity and spatial position varied systematically across trials, as we manipulated stimulus repetition and controlled for the participant's expectancy as to whether a stimulus would change on any given trial.

Before the fMRI experiments, we calibrated the psychometric functions of each individual so that during scanning we could present stimuli that balanced the participant's expectancy that pairs of stimuli would be different or not. Perceptually balanced psychometric functions help to minimize differential perceptual expectations for repeated stimuli that may have occurred in previous studies where there were more (perceptually) “different” trials than “same” trials (Murray and Wojciulik, 2004). During the mock scanner calibration session we estimated the perceptual identity levels and spatial position levels needed to balance the psychometric function, which were subsequently used for that individual's fMR-A session. This ensured that nearly half of the stimulus repetition conditions would be perceived as being a stimulus “change” and the others “no-change” (see Fig. 3*A*,*B*), while we assessed attention and adaptation interactions.

For the identity task, participants were instructed to attend to identity and ignore any changes to the spatial position between the two stimuli. For the spatial position task, participants were instructed to attend to spatial position and ignore any changes to identity between the two stimuli. Across participants, we counterbalanced the hand and which finger they used to make their responses. Before each block, the participants completed 20 practice trials with feedback to familiarize themselves with the procedure and stimuli. Each block took ∼25 min to complete, and breaks between blocks were given to reduce fatigue. Each participant was trained in a calibration session in the mock scanner followed by two fMR-A scanning sessions. The three sessions were run on separate days.

The identity and spatial position levels are defined as the physical stimulus difference in identity or spatial position, respectively, between a stimulus pair that leads to balanced discrimination performance. To achieve this we estimated the 50 and 75% perceptual levels (i.e., 50 and 75% correct discrimination) in Experiment 1 by fitting psychometric functions to participants' discrimination responses for each stimulus feature.

For identity, the physical stimulus difference was the morph difference between the two faces or two voices presented on a given trial. Seven morph differences were selected as follows. We first randomly selected with replacement a morph stimulus face/voice pair from the 21 face/voice stimuli available. We then selected the second morph stimulus from the remaining 20 stimuli that provided the desired morph difference. For example, suppose we randomly select a face stimulus that was a 35% morph between Face A and Face B. In this case, for a 10% morph difference, we would randomly select either the 25% or the 45% morph between Face A and Face B. For a 0% morph difference (i.e., same stimulus), we would select the same 35% morph between Face A and Face B. Across participants, we sampled the entire identity morph continuum for each face/voice pair. As expected, the morph differences varied slightly for each participant. On average, the seven physical morph differences were for voices: 0 (same), 11.3, 21.3, 32.5, 45.0, 55.0, and 66.3%, and for faces: 0 (same), 11.7, 22.1, 33.3, 45.7, 56.3, and 67.5%.

For spatial position, the physical stimulus difference was the relative spatial displacement between the two voices or the two faces presented on each trial. The seven spatial position differences were selected in an analogous manner as for identity. For the voice stimuli, the two stimuli were presented sequentially in the right or left auditory field separated by, on average, 0 (same), 2.9, 6.1, 9.0, 11.9, 14.8, and 17.5° along the azimuth. For the face stimuli, the center of the face images were presented sequentially in the right or left visual field (i.e., right or left half of the screen) separated by, on average, 0 (same), 9, 18, 27, 36, 45, or 54 pixels (∼45 pixels/° of visual angle). For both the face and voice stimuli, the stimuli occurred equally often in the right and left auditory/visual field.

We estimated the 50% identity level from participants' discrimination responses as follows. For each stimulus type, we first calculated the proportion different response as a function of morph difference, pooling across spatial position differences, based on the 42 trials for each of the seven morph differences (6 face/voice pairs × 7 spatial differences). We then fitted a cumulative Gaussian function to each participant's responses for each condition. For each of the two psychometric functions, we estimated the morph difference, which gave rise to 50% correct responses for the middle morph difference. The same procedure was used to estimate the 75% identity level and 50% and 75% spatial position levels, pooled across identity morph differences, also based on the 42 trials for each of the seven spatial differences (6 face/voice pairs × 7 morph differences).

Table 1 shows the estimated 50 and 75% identity and spatial position levels averaged across participants.

**Table 1.** Average stimulus level (and SEM) calculated during behavioral testing in a mock scanner used to determine individual participant stimulus levels for the subsequent fMRI experiments

	Experiment 1		Experiment 2	
	50%	75%	50%	80%
Voice				
Identity	36.5 (4.4)	49.1 (5.3)	48.9 (2.1)	67.0 (3.7)
Spatial/intensity	7.2 (1.4)	10.4 (1.2)	0.23 (0.02)	0.41 (0.04)
Face				
Identity	40.6 (3.1)	54.4 (3.4)		
Spatial	27.1 (2.7)	39.1 (3.6)		

Identity level was measured in percentage morph difference between the two stimuli. Spatial level was measured in pixel difference between the two faces, and simulated azimuth difference in degrees between two voices. Intensity level was measured in loudness difference (arbitrary unit) between the two pink noise elements of the acoustic object.

The four conditions resulting from the factorial combination of stimulus type (face, voice) and task (identity, spatial position) were run in separate blocks, counterbalanced across participants using a Latin square design. Each block consisted of 294 trials (6 face/voice pairs × 7 morph differences × 7 spatial differences) presented in random order. Each trial began with a white fixation cross presented at the center of the screen for 500 ms, followed by a blank period (black screen) for 1500 ms. The two stimuli were then presented sequentially for their duration, separated by a 500 ms black screen. The participants were instructed to respond by pressing a same or different key after the offset of the second stimulus.

##### fMRI procedure and experimental design.

On each fMR-A session, participants were presented with only face or voice stimuli, and they discriminated either identity or spatial position on four alternating runs (2 runs per attended feature). Both the order of stimulus type and the order of task were counterbalanced across participants using a Latin square design. Each run was ∼8 min in duration, and consisted of 54 trials (6 face/voice pairs × 3 identity levels × 3 spatial position levels). The three perceptual levels for each stimulus feature [0 (same trial), 50, and 75%] were individualized for each participant as described in the prior section, and were randomly interleaved within a run.

We used a sparse imaging protocol so that auditory stimuli could be presented during silent periods without scanner noise. This is a common auditory fMRI scanning paradigm so that the scanner noise does not mask or compete with the acoustic stimuli (Hall et al., 1999); although it is not required for the visual modality, we opted to keep the scanning paradigm the same throughout. The scanner transmitted a transistor–transistor logic (TTL) pulse every 8000 ms and acquired an MRI volume in 2000 ms after each pulse. Each trial sequence was triggered by the TTL pulse. The trial began with a 2000 ms blank screen, followed by a white fixation cross at the center of the screen for 500 ms. Then the first stimulus was presented for its duration, followed by a black screen for 500 ms (interstimulus interval), and then the second stimulus for its duration, with the stimulation trial ending with a white fixation cross. This white cross changed color from white to green 6000 ms after trial onset and remained on the screen for 1700 ms during which time the participants made their responses (Fig. 1*C*). There was a brief 300 ms blank period before a TTL pulse triggered the next trial. The MRI volume acquired on a given trial is timed to occur ∼4 s after the offset of the second stimulus to accommodate for the delay in the hemodynamic response (Fig. 1*C*,*D*).

Participants were instructed to respond by pressing the same or different key during the response period when the fixation cross was green. If they did not respond during this period, the trial was counted as an error. As for the mock-scanner experiments, across participants, we counterbalanced the hand and which finger they used to make their responses. Each auditory or visual fMR-A testing session lasted ∼1 h, which included structural scans if needed. In addition, during one of the scanning sessions we also acquired functional data during face (Schultz and Pilz, 2009) and voice (Belin et al., 2000) localizer runs to identify face- and voice-sensitive regions.

#### Experiment 2

##### Participants.

A separate group of 12 volunteers (7 females, age 28.5 ± 7.8 SD; 5 males age 24.4 ± 3.1 SD) participated in Experiment 2 conducted in the auditory modality. The number of participants was the same as those scanned for the auditory Experiment 1 to ensure the same statistical power across both experiments.

##### Stimuli.

The Experiment 2 stimuli consisted of the voice stimuli from Experiment 1 concatenated with a short noise burst (50 ms) at the end. Note that we treat the concatenated stimuli as a single complex acoustic object as there are no delays between the two components (with identity and loudness being intrinsic features of this object). For the noise bursts, we used MATLAB to synthesize acoustic pink-noise stimuli that varied in intensity. Pink noise has a 1/*f* power spectrum that is present in naturally occurring environmental sounds, containing high-power at low frequencies with an exponential decrease in power as frequency increases. To vary perceived loudness, we systematically manipulated the (physical) mean intensity of the pink noise (i.e., intensity averaged across the entire 50 ms duration). Like voice morph percentages, there were 21 levels of mean intensity of the pink noise ranging from 0.5 to 0.95 in 0.0214 steps (arbitrary normalized intensity unit). On each trial, we independently manipulated the voice identity and the concatenated noise stimulus intensity.

##### Perceptual calibration, fMRI procedure, and experimental design.

All participants were tested in a mock scanner calibration session followed by one fMR-A session on a separate day. The procedure for both sessions was the same as in Experiment 1, except that participants discriminated identity or loudness across two pairs of complex stimuli. For the identity task, participants were instructed to judge whether the identity of the two voices was the same while ignoring any changes to the noise stimulus. For the loudness task, participants were instructed to judge the loudness of the two noise stimuli while ignoring any changes in voice identity.

Similar to Experiment 1, the purpose of the perceptual calibration session was to estimate the 50% and 80% identity and loudness levels, which were subsequently used for the fMR-A session for each participant (Table 1). The two perceptual levels for voice identity were determined as in Experiment 1. For the perceptual level of loudness, the physical stimulus difference was the intensity difference between the two noise stimuli on a given trial. The 50% and 80% loudness levels were determined as follows. We first randomly selected a noise stimulus from the 21 possible stimuli. The intensity of the second noise was selected to give a balanced behavioral response function. For Experiment 2, the voice morph differences were as follows: 0, 15, 30, 45, 60, 75, and 90%; and the intensity differences were as follows: 0, 0.0645, 0.129, 0.1935, 0.258, 0.3225, and 0.387 arbitrary units. There were 294 trials for each attended feature (6 voice pairs × 7 morph differences × 7 intensity differences) were run in a random order. The auditory stimuli were always presented at 0° azimuth in virtual acoustic space (i.e., straight ahead).

In the fMR-A session in Experiment 2, participants discriminated either identity or loudness in four alternating runs (2 runs per attended feature). The task order was counterbalanced across participants. As in Experiment 1, we systematically manipulated the magnitude of identity or intensity changes on each trial within a run based on each participant's estimated 50% and 80% perceptual level for each stimulus feature. Before testing, participants were given 20 practice trials with feedback. Each run was ∼8 min in duration and consisted of 54 trials (6 voice pairs × 3 identity levels × 3 loudness levels) run in a random order (for an illustration of the trial sequence, see Fig. 1*D*). The three perceptual levels were 0, 50, and 80%. The scanning session lasted ∼1 h, which included structural and functional scans including a voice localizer (Belin et al., 2000).

##### Image acquisition.

For all participants in Experiments 1 and 2, anatomical T1-weighted images and functional T2*-weighted gradient-recall echo planar images (EPIs) were acquired from a 3-Tesla Philips Intera Achieva MR scanner using a Philips 8-channel head coil. The higher resolution T1-weighted structural image consisted of 150 slices and took ∼5 min to acquire. The parameters of the structural scan were as follows: repetition time (TR) = 9.6 ms, echo time (TE) = 4.6 ms, flip angle = 8°. The field-of-view (FOV) was 249 × 240 × 180 mm^3^ with a matrix size of 216 × 208 pixels. Each voxel was 0.87 × 0.87 × 1.2 mm^3^ in size. The T2*-weighted EPI functional images consisted of 28 axial slices acquired from the bottom to the top of the head. The parameters of the EPI scans were as follows: acquisition time = 2.0 s, TR = 8.0 s, TE = 30 ms, flip angle = 90°. The FOV was 192 × 192 × 125.5 mm^3^ with a matrix size of 64 × 62 pixels. Each voxel was 3 × 3 × 4 mm^3^ in size, with a 0.5 mm gap between slices. We use sensitivity encoding (SENSE) with factor = 2 to increase the signal-to-noise ratio of the functional images. For each participant, a total of 54 functional images were acquired in each run. Before each functional run, four “dummy” scans were acquired to allow for equilibration of the T1 signal.

##### fMRI preprocessing.

We used FSL (Jenkinson et al., 2012) for all fMRI data processing and analyses. The data acquired for the auditory and visual stimuli were analyzed separately. For each dataset, we removed the first volume from each run and concatenated all four runs into a single time series. We then extracted each participant's brain from the skull using BET, normalized the intensity of the resulting functional images, and spatially coregistered the images to that participant's structural scan. The images were spatially smoothed with a 5 mm full-width at half-maximum Gaussian kernel to improve the signal-to-noise ratio. Last, we applied a high-pass filter with a cutoff of 432 s to remove low-frequency drifts in the signal.

##### Face and voice regions-of-interest.

To identify face-sensitive areas, we used the face localizer from Schultz and Pilz (2009). Briefly, this localizer consisted of fixations, static faces, static phase-scrambled faces, dynamic faces, and dynamic phase-scrambled faces. These conditions were run in a block design, with every condition preceded by each condition equally often (i.e., history-matched blocks). Participants performed a one-back matching task. The face localizer lasted ∼7.5 min, and we used a TR = 1.92 s. To identify voice-sensitive areas, we used the voice localizer from Belin et al. (2000). Briefly, there were 20 × 8 s vocal sounds, 20 × 8 s non-vocal sounds (industrial, environmental, and animal sounds) and 20 silent periods. The stimuli were presented in a fixed intermixed order optimized for the contrast vocal > non-vocal sounds. Participants performed a fixation-change detection task. The duration of the voice localizer was 10 min and we used a TR = 10 s.

**Figure 2.**
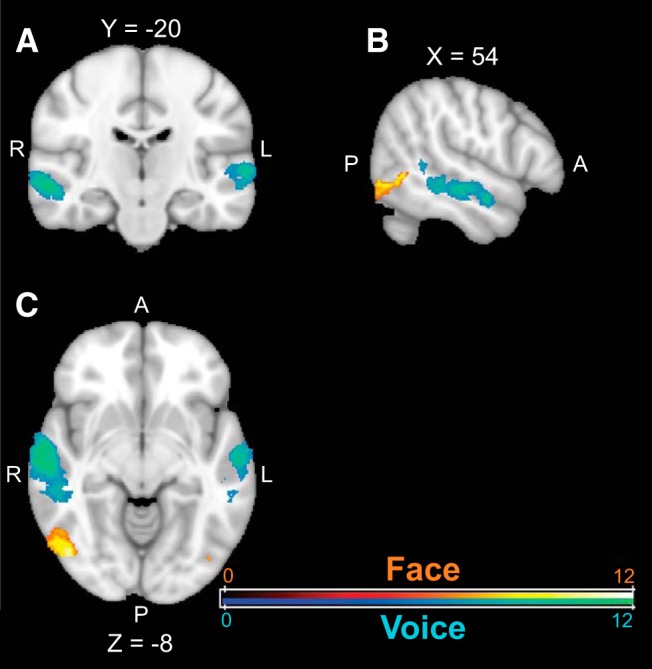
Voice and face localizer ROIs. Averaged map of face (red) and voice (blue) areas in the coronal (***A***), horizontal (***B***), and sagittal (***C***) views across 12 participants who participated in Experiment 1. Only the areas that are activated in more than half of the participants (*N* > 6) are shown.

Functional data from the localizer runs were preprocessed as described above, and spatially coregistered to each participant's structural scan. Following this, β images for the different stimulus conditions were calculated using procedures described by Schultz and Pilz (2009) and Belin et al. (2000); these images were used in different contrasts to functionally localize face- and voice-sensitive areas. The face regions-of-interest (ROIs) for each participant were defined as voxels from the contrast (static and dynamic faces) > (static and dynamic phase-scrambled faces) with *Z* > 5.3 and limited to be within the anatomically-defined temporal lobe. Similarly, the voice ROIs for each participant were defined as voxels from the contrast vocal > non-vocal sounds with *Z* > 5.3 and limited to be within the temporal lobe. Face and voice ROIs are shown in Figure 2. We used standard human atlases in FSL for the temporal lobe (Harvard-Oxford Cortical Structural Atlas and the Juelich Histological Atlas) and spatially coregistered this region to each participant's structural scan.

##### Statistical analyses.

On separate functional runs, participants discriminated changes to one stimulus feature (e.g., identity) of face or voice pairs, while ignoring changes to another feature (e.g., spatial position). The magnitude of physical changes for both stimulus features was systematically varied. For both experiments, we compared the magnitude of BOLD responses in face and voice ROIs (see above) across different experimental conditions. To estimate the magnitude of BOLD responses for each condition, we performed a first-level fixed-effects general linear model analysis using FSL's FEAT function. For each participant and each stimulus type, we constructed two design matrices depending on whether we varied the identity level or the spatial position/loudness level. Each design matrix was constructed as follows. There were six regressors-of-interest to reflect the six experimental conditions [2 tasks × 3 perceptual levels (either identity or spatial position/loudness)]. Because we used sparse imaging, each fMRI time point in the design matrix reflects the BOLD response to the stimulus pair presented on that trial. In addition, the six movement parameters (roll, yaw, pitch, and 3 translation terms) calculated from the motion correction were included in the design matrix as regressors-of-no-interest. A linear combination of the regressors was fitted to the BOLD signal to calculate the parameter estimate for each regressor. The parameter estimate for each experimental condition was extracted from each voxel in the respective individually-localized face or voice ROIs and averaged.

For Experiment 1, the proportion different responses and the mean parameter estimates were submitted to two separate within-subjects ANOVAs with stimulus type (face, voice), task (identity, spatial position) and either identity level or spatial position level (0, 50, and 75% for both) as repeated measures. For Experiment 2, the data were also submitted to separate within-subjects ANOVAs with task (identity, loudness) and either identity level or loudness level (0, 50, and 80% for both) as repeated measures. We also conducted a priori trend analyses to detect polynomial trends in the data (e.g., linear trends). For all analyses, we used a significance threshold of *p* = 0.05. All analyses were conducted using MATLAB and/or SPSS v23.

## Results

In this study, we conducted several attentional manipulations within the context of conditions eliciting systematic fMR-A repetition suppression. As an overview, in Experiment 1 in two sensory modalities (auditory and visual) participants were presented with pairs of morphed faces or voices while we measured how the BOLD response changed as a function of the perceptual differences between the stimuli. Here, the participants either attended to identity or spatial position. In Experiment 2, we tested the influence of attention to acoustic features of the complex auditory objects by measuring fMR-A effects while participants listened to pairs of voices and discriminated their identity or the loudness of a noise burst presented immediately after the vocalization.

First, we overview our predictions and how the neuroimaging results are analyzed to test them: fMR-A effects are strongest for repetition of identical stimuli, thus we expected that the mean parameter estimate in face- and voice-sensitive ROIs would increase in response to the second stimulus as we morphed voice or face identity levels away from the identity presented as a first stimulus. This serves as the fMR-A stimulus-related adaptation function. If attention only affects gain during fMR-A and not selectivity, we expect that the mean parameter estimate in these ROIs would increase by equal amounts at each identity level when participants discriminated identity compared with when they discriminated spatial position (Experiment 1) or loudness (Experiment 2). By comparison, if attention affects neural selectivity, we expect that the mean parameter estimate would increase differentially at each identity level between tasks, evident as an interaction between task and identity level. Data for both experiments reported below are freely available at the Open Science Framework (https://osf.io/dxevz/).

### Experiment 1

#### Attention to identity

**Figure 3.**
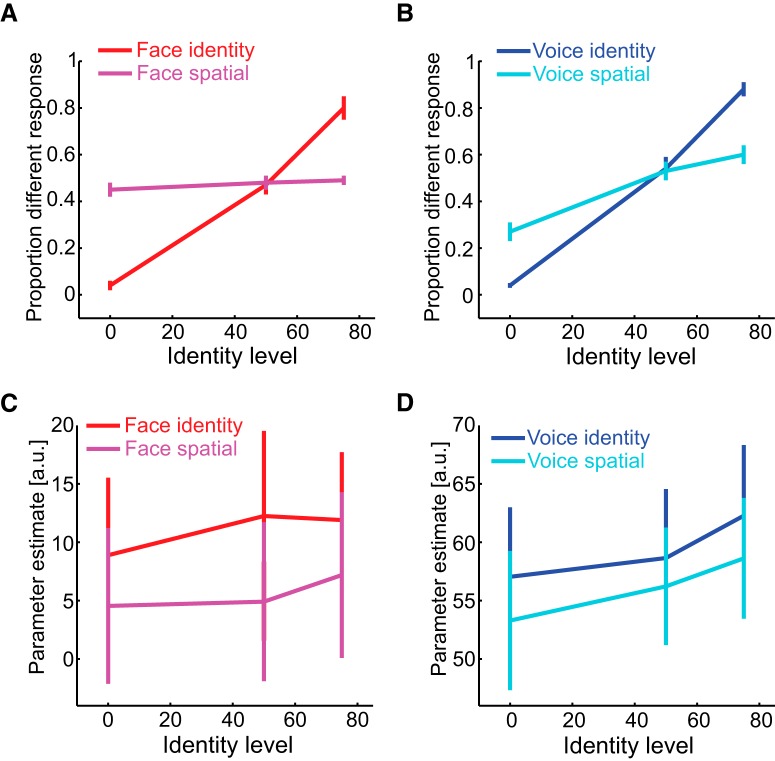
Attention to voice or face identity while ignoring stimulus spatial location. Behavioral (***A***, ***B***) and fMRI (***C***, ***D***) results from Experiment 1 when stimulus identity is varied. ***A***, Perceptual discrimination of face identity (red) and spatial position (magenta) as face identity varies. ***B***, Perceptual discrimination of voice identity (blue) and spatial position (cyan) as voice identity varies. ***C***, Parameter estimates from the face ROI when discriminating face identity (red) and spatial position (magenta) as face identity varies. ***D***, Parameter estimates from the voice ROI when discriminating voice identity (blue) and spatial position (cyan) as voice identity varies. The error bars denote SEM.

Participants' ability to discriminate identity and spatial position as a function of identity level for faces and voices is shown in Figure 3, *A* and *B*. As expected, participants' proportion different response increased as identity level increased for both faces and voices (i.e., as the morph difference between the two stimuli increased) but only when they attended to and discriminated identity (red and blue lines) rather than spatial position (magenta and cyan lines). These observations are reflected by a significant interaction between task and identity level for faces (*F*_(2,22)_ = 121.474, *p* < 0.001, η_p_^2^ = 0.917) and for voices (*F*_(2,22)_ = 26.572, *p* < 0.001, η_p_^2^ = 0.707). There was a main effect of identity level (*F*_(2,22)_ = 121.053, *p* < 0.001, η_p_^2^ = 0.917), but not task (*F*_(1,11)_ = 1.389, *p* = 0.263, η_p_^2^ = 0.112) for faces, and a main effect of identity level (*F*_(2,22)_ = 212.264, *p* < 0.001, η_p_^2^ = 0.951), but not task (*F*_(1,11)_ = 0.261, *p* = 0.600, η_p_^2^ = 0.026) for voices. The behavioral data in this and the subsequent experiment validate the perceptual levels estimated during the calibration sessions.

During fMRI, participants attended voice or face identity and Figure 3, *C* and *D*, shows the mean parameter estimate as a function of stimulus type, task, and identity level. Participants' mean parameter estimate in face and voice ROIs increased as identity level increased, commensurate with release from adaptation (main effect of identity level: *F*_(2,22)_ = 5.151, *p* = 0.015, η_p_^2^ = 0.319; significant linear trend: *F*_(1,11)_ = 6.912, *p* = 0.023, η_p_^2^ = 0.386). We also found that attention to identity increased the mean parameter estimate in both ROIs (main effect of task: *F*_(1,11)_ = 5.329, *p* = 0.04, η_p_^2^ = 0.33). Importantly, we found no significant interaction between task and identity level and no significant three-way interaction (all *F* values < 1.043) indicative of only increases in gain as a function of attention. Finally, there was a significant main effect of stimulus type (*F*_(1,11)_ = 34.484, *p* < 0.001, η_p_^2^ = 0.758), but no interactions between stimulus type and task or identity level (all *F* values < 0.525).

#### Attention to spatial position

**Figure 4.**
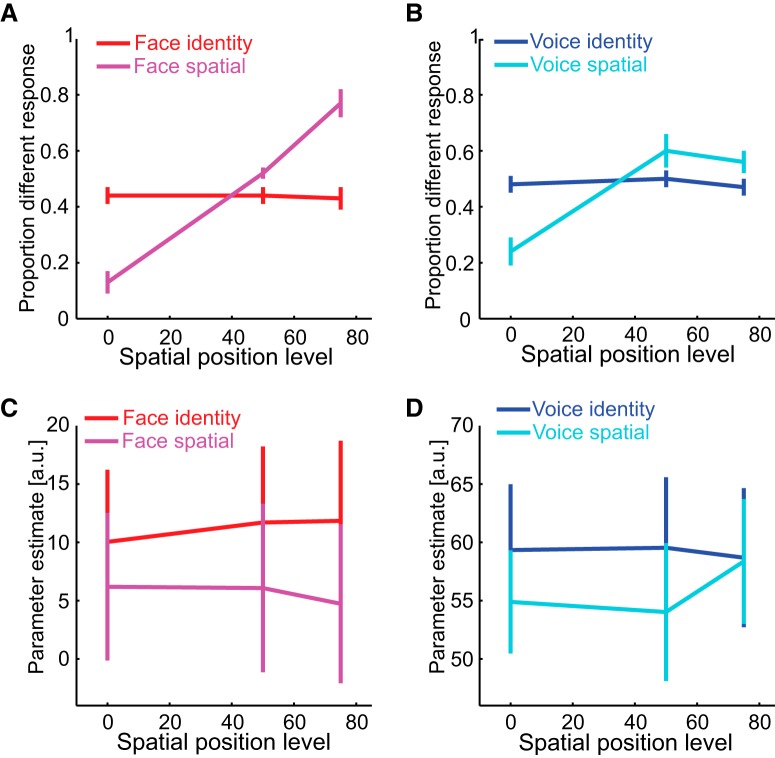
Attention to spatial location while ignoring voice or face identity. Behavioral (***A***, ***B***) and fMRI (***C***, ***D***) results from Experiment 1 when stimulus position is varied. ***A***, Perceptual discrimination of face identity (red) and spatial position (magenta) as face position varies. ***B***, Perceptual discrimination of voice identity (blue) and spatial position (cyan) as voice position varies. ***C***, Parameter estimates from the face ROI when discriminating face identity (red) and spatial position (magenta) as face position varies. ***D***, Parameter estimates from the voice ROI when discriminating voice identity (blue) and spatial position (cyan) as voice position varies. The error bars denote SEM.

Participants' behavioral ability to discriminate spatial position for faces and voices is shown in Figure 4, *A* and *B*. Similar to the first analysis, participants' proportion different responses increased as spatial position level increased for both stimulus types (i.e., as the spatial displacement between the two stimuli increased) only when they attended to and discriminated spatial position (magenta and cyan lines) but not identity (red and blue lines). These observations are reflected in a significant interaction between task and spatial position level for faces (*F*_(2,22)_ = 64.981, *p* < 0.001, η_p_^2^ = 0.855) and for voices (*F*_(2,22)_ = 15.095, *p* < 0.001, η_p_^2^ = 0.578). There was a main effect of spatial position level (*F*_(2,22)_ = 74.950, *p* < 0.001, η_p_^2^ = 0.872), but not task (*F*_(1,11)_ = 1.347, *p* = 0.270, η_p_^2^ = 0.109) for faces, and a main effect of spatial position level (*F*_(2,22)_ = 16.401, *p* < 0.001, η_p_^2^ = 0.599), but not task (*F*_(1,11)_ = 0.278, *p* = 0.608, η_p_^2^ = 0.025) for voices.

As for the fMRI results during attention to spatial location, the mean parameter estimate as a function of stimulus type, task and spatial position level is shown in Figure 4, *C* and *D*. The mean parameter estimate was greater when the participants attended to and discriminated identity compared with spatial position (main effect of task: *F*_(1,11)_ = 5.714, *p* = 0.039, η_p_^2^ = 0.342). Moreover, unlike the previous analysis, their mean parameter estimate did not significantly increase with spatial position level, meaning that there was no spatial adaptation effect in face- or voice-sensitive cortex. There were also no interactions (all *F* values < 1.0). Finally, there was a main effect of stimulus type (*F*_(2,22)_ = 33.656, *p* < 0.001, η_p_^2^ = 0.754). These results are expected because face and voice ROIs are more involved in processing face or voice features than spatial location. Furthermore, although attention to spatial position increased the gain of neural responses in these ROIs, it did not change the selectivity of these responses.

### Experiment 2

#### Attention to identity

**Figure 5.**
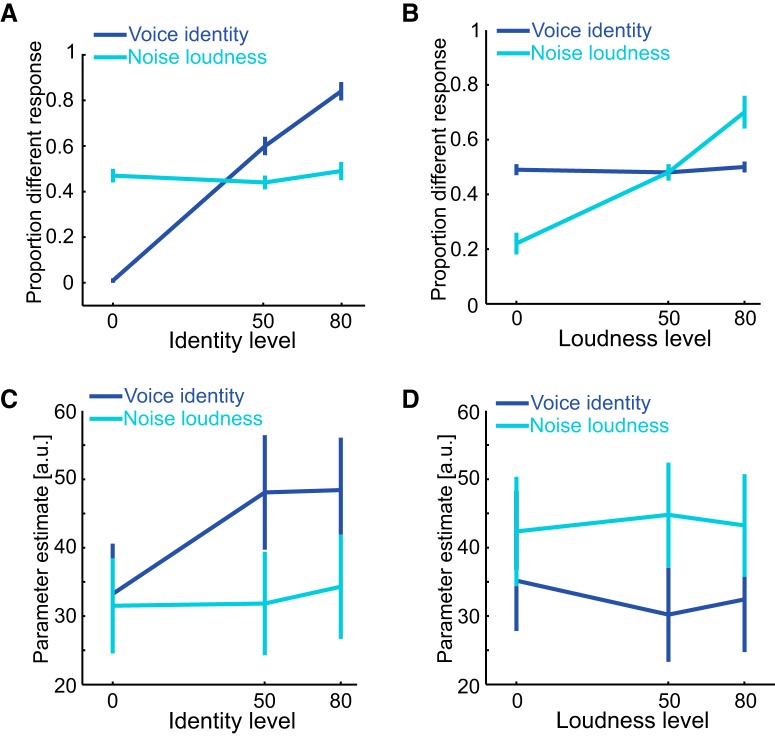
Attention to voice identity versus loudness. Behavioral and fMRI results from Experiment 2 where stimulus identity and loudness are varied. Perceptual discrimination of voice identity (blue) and sound loudness (cyan) as voice identity varies (***A***) and loudness varies (***B***). Parameter estimates from the voice ROI when discriminating voice identity (blue) and loudness (cyan) as voice identity varies (***C***) and loudness varies (***D***). The error bars denote SEM.

Participants' ability to discriminate identity and loudness as a function of identity level is shown in Figure 5*A*. As expected, the proportion different responses increased as identity morph distance increased only when the participants attended to and discriminated identity (blue line) but not loudness (cyan line). These observations are reflected in a significant interaction between task and identity level (*F*_(2,22)_ = 225.525, *p* < 0.001, η_p_^2^ = 0.953). There was a main effect of identity level (*F*_(2,22)_ = 123.443, *p* < 0.001, η_p_^2^ = 0.918), but not task (*F*_(1,11)_ = 0.772, *p* = 0.399, η_p_^2^ = 0.066).

The mean fMRI parameter estimate as a function of task and identity level is shown in Figure 5*C*. As found in Experiment 1, participants' mean parameter estimate in voice ROIs increased as identity level increased, commensurate with release from adaptation (main effect of identity level: *F*_(2,22)_ = 18.006, *p* < 0.001, η_p_^2^ = 0.621; significant linear trend: *F*_(1,11)_ = 42.5, *p* < 0.001, η_p_^2^ = 0.795). In addition, there was a main effect of task (*F*_(1,11)_ = 5.710, *p* = 0.036, η_p_^2^ = 0.342). However in contrast to Experiment 1, there was a significant interaction between task and identity level (*F*_(2,22)_ = 18.004, *p* < 0.001, η_p_^2^ = 0.621) indicative of a change in selectivity during attention to voice identity. To interrogate this interaction further, we analyzed main effects of identity level separately for each task. We found that the mean fMRI parameter estimate varied as a function of identity level for the identity task (*F*_(2,22)_ = 30.16, *p* < 0.001, η_p_^2^ = 0.733; linear trend: *F*_(1,11)_ = 88.264, *p* < 0.001, η_p_^2^ = 0.889; quadratic trend: *F*_(1,11)_ = 9.512, *p* = 0.01, η_p_^2^ = 0.464) but not for the loudness task (*F*_(2,22)_ = 1.281, *p* = 0.298, η_p_^2^ = 0.104). Overall, our findings suggest that attention to voice identity while ignoring loudness increases both the gain and selectivity of neural responses in the voice ROIs during fMR-A.

#### Attention to loudness

Participants' ability to discriminate identity and loudness as a function of loudness level is shown in Figure 5*B*. Their proportion of different response increased as loudness level increased (i.e., as the intensity difference between the two noise component of the complex stimuli increased) only when they attended to and discriminated loudness (cyan line) but not identity (blue line). These observations are reflected in a significant interaction between task and loudness level (*F*_(2,22)_ = 29.936, *p* < 0.001, η_p_^2^ = 0.731). There was a main effect of identity level (*F*_(2,22)_ = 37.498, *p* < 0.001, η_p_^2^ = 0.773), but not task (*F*_(1,11)_ = 0.846, *p* = 0.378, η_p_^2^ = 0.071).

The mean fMRI parameter estimate as a function of task and loudness level is shown in Figure 5*D*. Similar to Experiment 1, participants' mean parameter estimate was greater when they attended to and discriminated loudness compared with identity (main effect of task: *F*_(1,11)_ = 5.872, *p* = 0.034, η_p_^2^ = 0.348). As expected, there was no main effect of loudness level (*F* < 1), suggesting no clear adaptation function for loudness level in voice-sensitive cortex. However, unlike the analogous analysis from Experiment 1, there was a significant interaction between task and loudness level (*F*_(2,22)_ = 4.430, *p* = 0.024, η_p_^2^ = 0.287). This interaction was driven by a quadratic trend (*F*_(1,11)_ = 5.314, *p* = 0.042, η_p_^2^ = 0.326; linear trend: *F*_(1,11)_ = 2.890, *p* = 0.117, η_p_^2^ = 0.208; Fig. 5*D*). Thus although the voice ROI is insensitive to changes in noise intensity, attention to the complex stimulus features including loudness nonetheless moderated the selectivity of neural responses within the ROI.

Finally, to assess whether the effects on adaptation of attending to different stimulus features were significantly different from each other in Experiments 1 and 2, we performed an additional three-way mixed ANOVA on the fMRI parameter estimates measured when participants were presented with auditory stimuli and we varied the stimulus identity. Here we entered Experiment as a between-subjects factor (Experiment 1, Experiment 2), along with within-group factors of task (attend identity, attend other: position or loudness) and stimulus level (0, 50, 75/80%). The principal finding from this analysis was the significant three-way interaction (*F*_(2,44)_ = 10.882, *p* < 0.001, η_p_^2^ = 0.331) that reflects that the effect of the focus of attention on adaptation effects is significantly different between the two experiments in the auditory modality. This interaction is consistent with the gain effects we observed in Experiment 1 and the selectivity (and gain) effects we observed in Experiment 2.

## Discussion

The results show that what we attend to in our environment interacts with brain adaptation in distinctly different ways, impacting on cortical response gain or selectivity during adaptation. The findings reconcile prior results showing either attention-related gain (Altmann et al., 2008) or changes in selectivity (Murray and Wojciulik, 2004) during adaptation. Attentional gain modulation during adaptation has most commonly been observed, and to date is the most parsimonious explanation for such interactions (for review, see Summerfield and de Lange, 2014), which we also see here under certain conditions. However, our combined results both challenge the notion that attentional influences during fMR-A only affect response gain, and they clarify the conditions under which attention and adaptation interact to change selectivity.

In the first experiment, with selective attention to identity versus spatial position, attention works additively with adaptation. The effects were remarkably similar in auditory or visual modalities, in voice- or face-sensitive cortex, respectively. Here, participants attended to the identity of voices/faces while ignoring the spatial location of the stimuli, or vice versa. Because the voice/face localizer ROIs were selected to identify areas processing these features, attention to stimulus features versus their spatial location in this case selects predominantly separate neural populations. For instance, temporal lobe voice-sensitive areas do not overlap with cortical regions involved in sound localization (Belin et al., 2000; Zatorre et al., 2002), although spatial operations are known to be more broadly distributed in auditory cortex than previously thought (Ortiz-Rios et al., 2017). Thereby, voice- and face-sensitive regions will adapt to these stimulus features and reduce their responses more to repetition of stimuli with similar features, as a form of neural selection. In these regions, and independent of the level of adaptation, attention amplifies and counteracts the adapted neural signal by a constant multiplicative factor. The resulting fMR-A interaction is a constant change in gain.

By contrast, in the second experiment, attention to identity results in disproportionately greater enhancement for conditions during which there is less adaptation in voice-sensitive cortex; this interaction between attention and adaptation is consistent with increases in neural selectivity (Murray and Wojciulik, 2004). Although intensity is not an optimal feature for voice-sensitive cortex, seen as weak to no adaptation effects to sound intensity, attention to identity or loudness interacted in ways that were different from the effects seen during attention to identity or spatial location. Adaptation, as in Experiment 1, again acts as a neural selection process tuned to identity. However, in Experiment 2, attention needs to boost the activity of a subpopulation responding to voice content over sound intensity. Specifically, in trials where both voices presented are equal (identity difference = 0%), there is full adaptation and attention to identity negligibly boosts the signal. However, in trials where two voices differ by 50%, some neurons that are specifically tuned for identity adapt less because of the different features being processed in succession. Upon this, we see that attention selectively boosts the reduced adaptation response to these stimuli. The effect of attention in this case, will therefore, be dependent on the level of adaptation, but rather than a “constant gain change” as we see in the first experiment, there is an interaction between adaptation and attention.

We now consider how these results can be more formally understood within the broader literature on attentional interactions with different types of predictive signals. SSA, one of the processes that we manipulated here, is thought to result from subcortical and cortical inhibitory interneuron influences on pyramidal neurons, typically based on neuronal data modeled as a decrease in neuronal response gain without a change in selectivity (Parras et al., 2017). Neurophysiological data on goal-directed attention has led to computational models accounting for both attentional gain and changes in selectivity. For instance, in the normalization model of attention by Reynolds and Heeger (2009) two mechanisms lead to attentional enhancement and a change in selectivity. The model is as follows: *R* = *E*/*N* where the neural response (*R*) results from two processes:attentional enhancement (*E*), which is the product of the stimulus–response field (*Sf*) and the attention field (*Af*) where attention is directed; and divisive normalization (*N*) created by the suppressive surround for features that fall outside the attentional field and need to be ignored. Typically changes in selectivity occur when there is “biased competition”, such as when competing features that spatially overlap are either being attended to or ignored (Desimone, 1998).

Murray and Wojciulik (2004) observed and modeled an increase in selectivity during fMR-A as a function of attention. First, they modeled the greater adaptation for more similar stimulus features, which we depict here as follows: â(*s*), where (*s*) is the stimulus-driven response or tuning curve and â is the effect of adaptation as a function of tuning. Then they modeled the multiplicative effect of attention (*g*) on the adaptation function as follows: *g*â[g(*s*)]. Note that the nesting of the attentional gain (*g*) with both (â) and (*s*) is required for a change in fMR-A selectivity: the greater effect of attention on stimuli that produce less adaptation. Integrating the effects of adaptation into the normalization model of attention can be done as follows: *R* = [*E* × (Af*â)]/*N*. As before, *E* is attentional enhancement, equivalent to *g*(*s*) in the adaptation model, and *Af* × â reflects attention interacting with the level of adaptation, equivalent to *g*â in the adaptation model.

Other prominent models that are relied on by the research community to explain attentional influences are those based on predictive coding. In the predictive coding model of brain function (Friston, 2005; Rao, 2005; Bastos et al., 2012), neurons compute the difference between sensory input and prior predictions, resulting in a prediction error passed from lower to higher hierarchical processing stages. Attention, in this setting, is a top-down influence that weighs the flow of prediction errors: prediction errors from neurons responding to attended object features are given more weight than those to ignored features. Mathematically, the weight is computed as precision (inverse of the SD: σ) of the distribution of prediction errors. Within this framework the neural response can be summarized as follows: $R=\frac{\left(\text{y}-\hat{y}\right)}{\sigma }$. Here (*y* − *ŷ*) is the prediction error and σ is the SD of prediction errors. The change in precision (σ) is equivalent to the normalization term (*N*) in the Reynolds and Heeger (2009) model. This term can also change the gain of attentional enhancement (1/σ is equivalent to *g* in the adaptation or the attentional field *Af* in the normalization models above). Thus considering the effects of adaptation on selectivity, the modified predictive coding model accounting for adaptation is as follows: $R=\frac{\left(\text{y}-\hat{y}\right)}{\sigma \left(\hat{a}\right)}$, where â, as above, is the adaptation function and σ(â) denotes the dependence of the SD (and precision) on the level of adaptation as a function of tuning.

Although, as we have considered, the predictive coding framework can formalize adaptation and attention influences, it is important to consider the distinctions between different types of predictive signals. For instance, typically within this framework sensory input is processed in relation to a (prior) expectation. However, expectancy effects (controlled in our experiment), partially segregate from SSA effects, are more accessible to consciousness, and thus might interact with attention differently (Summerfield and Egner, 2009; Kok et al., 2012a). Expectation suppression occurs when a repetition is expected, and when violated results in an expectation prediction error. Attention interacting with expectancy manipulations can result in different forms of fMRI-based changes in selectivity (Larsson and Smith, 2012; attentional dampening or sharpening during repetition expectation: Kok et al., 2012a; de Lange et al., 2018). However, even here expectancy effects sometimes only result in gain changes. Namely, Vinken et al. (2018) used the face repetition and expectancy manipulation paradigm from Summerfield et al. (2008) but found no effect of repetition expectancy on neuronal local field potentials in face-sensitive areas in nonhuman primates. Whereas prior work on interactions between adaptation and attention contained a higher proportion of perceived stimulus differences during repetition, which may have influenced participants' expectancy and thus affected selectivity (Murray and Wojciulik, 2004), in our experiment we minimized differences in perceptual expectations individually (balanced psychometric functions where perceived stimulus repetition or change occur with equal probability). Therefore, our study aimed to clarify SSA effects interacting with attention. In this regard, the conditions under which gain and selectivity changes occur under expectancy manipulations could benefit from further study to identify common mechanistic principles.

In conclusion, the properties of the stimulus to which attention is directed appear to determine whether attention in interaction with adaptation effects solely modulates the gain of adapted signals or also affects selectivity. We find that adaptation induced gain changes are prominent during tasks where the observer's attention is directed to features processed by largely segregated neural populations (e.g., spatial vs object features). By contrast, changes in selectivity occur when attention is directed to competing stimulus features processed by neural populations that overlap to a greater extent (e.g., voice identity and sound intensity). Thus, attention plays a multifaceted role in how adaptation induced gain and selectivity changes affect the flow of information in the brain.

## References

[B1] Altmann CF, Henning M, Döring MK, Kaiser J (2008) Effects of feature-selective attention on auditory pattern and location processing. Neuroimage 41:69–79. 10.1016/j.neuroimage.2008.02.013 18378168

[B2] Barlow HB, Foldiak P (1989) Adaptation and decorrelation in the cortex. In: The computing neuron (Durnin R, Miall C, Mitchison GJ, eds), pp 54–72. Wokingham, UK: Addison-Wesley.

[B3] Barron HC, Dolan RJ, Behrens TE (2013) Online evaluation of novel choices by simultaneous representation of multiple memories. Nat Neurosci 16:1492–1498. 10.1038/nn.3515 24013592 PMC4001211

[B4] Barron HC, Vogels TP, Emir UE, Makin TR, O'Shea J, Clare S, Jbabdi S, Dolan RJ, Behrens TE (2016) Unmasking latent inhibitory connections in human cortex to reveal dormant cortical memories. Neuron 90:191–203. 10.1016/j.neuron.2016.02.031 26996082 PMC4826438

[B5] Bastos AM, Usrey WM, Adams RA, Mangun GR, Fries P, Friston KJ (2012) Canonical microcircuits for predictive coding. Neuron 76:695–711. 10.1016/j.neuron.2012.10.038 23177956 PMC3777738

[B6] Bekinschtein TA, Dehaene S, Rohaut B, Tadel F, Cohen L, Naccache L (2009) Neural signature of the conscious processing of auditory regularities. Proc Natl Acad Sci U S A 106:1672–1677. 10.1073/pnas.0809667106 19164526 PMC2635770

[B7] Belin P, Zatorre RJ, Lafaille P, Ahad P, Pike B (2000) Voice-selective areas in human auditory cortex. Nature 403:309–312. 10.1038/35002078 10659849

[B8] Brainard DH (1997) The psychophysics toolbox. Spat Vis 10:433–436. 10.1163/156856897X00357 9176952

[B9] Clifford CW, Wenderoth P, Spehar B (2000) A functional angle on some after-effects in cortical vision. Proc Biol Sci 267:1705–1710. 10.1098/rspb.2000.1198 12233765 PMC1690741

[B10] de Lange FP, Heilbron M, Kok P (2018) How do expectations shape perception? Trends Cogn Sci 22:764–779. 10.1016/j.tics.2018.06.002 30122170

[B11] Desimone R (1998) Visual attention mediated by biased competition in extrastriate visual cortex. Philos Trans R Soc Lond B Biol Sci 353:1245–1255. 10.1098/rstb.1998.0280 9770219 PMC1692333

[B12] Eger E, Henson RN, Driver J, Dolan RJ (2004) BOLD repetition decreases in object-responsive ventral visual areas depend on spatial attention. J Neurophysiol 92:1241–1247. 10.1152/jn.00206.2004 15056686

[B13] Friston K (2005) A theory of cortical responses. Philos Trans R Soc Lond B Biol Sci 360:815–836. 10.1098/rstb.2005.1622 15937014 PMC1569488

[B14] Goffaux V, Hault B, Michel C, Vuong QC, Rossion B (2005) The respective role of low and high spatial frequencies in supporting configural and featural processing of faces. Perception 34:77–86. 10.1068/p5370 15773608

[B15] Grill-Spector K, Kushnir T, Edelman S, Avidan G, Itzchak Y, Malach R (1999) Differential processing of objects under various viewing conditions in the human lateral occipital complex. Neuron 24:187–203. 10.1016/S0896-6273(00)80832-6 10677037

[B16] Grill-Spector K, Henson R, Martin A (2006) Repetition and the brain: neural models of stimulus specific effects. Trends Cogn Sci 10:14–23. 10.1016/j.tics.2005.11.006 16321563

[B17] Hall DA, Haggard MP, Akeroyd MA, Palmer AR, Summerfield AQ, Elliott MR, Gurney EM, Bowtell RW (1999) “Sparse” temporal sampling in auditory fMRI. Hum Brain Mapp 7:213–223. 10.1002/(SICI)1097-0193(1999)7:3<213::AID-HBM5>3.0.CO;2-N 10194620 PMC6873323

[B18] Jenkinson M, Beckmann CF, Behrens TE, Woolrich MW, Smith SM (2012) FSL. Neuroimage 62:782–790. 10.1016/j.neuroimage.2011.09.015 21979382

[B19] Kawahara H, Morise M (2011) Technical foundations of TANDEM-STRAIGHT, a speech analysis, modification and synthesis framework. Sadhana 36:713–727. 10.1007/s12046-011-0043-3

[B20] Kawahara H, Morise M, Takahashi T, Nisimura R, Irino T, Banno H (2008) Tandem-STRAIGHT: a temporally stable power spectral representation for periodic signals and applications to interference-free spectrum, F0, and aperiodicity estimation. In: IEEE international conference on acoustics, speech and signal processing, 2008, pp 3933–3936. Piscataway, NJ: IEEE.

[B21] Kok P, Jehee JF, de Lange FP (2012a) Less is more: expectation sharpens representations in the primary visual cortex. Neuron 75:265–270. 10.1016/j.neuron.2012.04.034 22841311

[B22] Kok P, Rahnev D, Jehee JF, Lau HC, de Lange FP (2012b) Attention reverses the effect of prediction in silencing sensory signals. Cereb Cortex 22:2197–2206. 10.1093/cercor/bhr310 22047964

[B23] Larsson J, Harrison SJ (2015) Spatial specificity and inheritance of adaptation in human visual cortex. J Neurophysiol 114:1211–1226. 10.1152/jn.00167.2015 26063774 PMC4725118

[B24] Larsson J, Smith AT (2012) fMRI repetition suppression: neuronal adaptation or stimulus expectation? Cereb Cortex 22:567–576. 10.1093/cercor/bhr119 21690262 PMC3278317

[B25] Malmierca MS, Anderson LA, Antunes FM (2015) The cortical modulation of stimulus-specific adaptation in the auditory midbrain and thalamus: a potential neuronal correlate for predictive coding. Front Syst Neurosci 9:19. 10.3389/fnsys.2015.00019 25805974 PMC4353371

[B26] Maunsell JH, Treue S (2006) Feature-based attention in visual cortex. Trends Neurosci 29:317–322. 10.1016/j.tins.2006.04.001 16697058

[B27] Murray SO, Wojciulik E (2004) Attention increases neural selectivity in the human lateral occipital complex. Nat Neurosci 7:70–74. 10.1038/nn1161 14647291

[B28] Okamoto H, Stracke H, Wolters CH, Schmael F, Pantev C (2007) Attention improves population-level frequency tuning in human auditory cortex. J Neurosci 27:10383–10390. 10.1523/JNEUROSCI.2963-07.2007 17898210 PMC6673146

[B29] Ortiz-Rios M, Azevedo FAC, Kuśmierek P, Balla DZ, Munk MH, Keliris GA, Logothetis NK, Rauschecker JP (2017) Widespread and opponent fMRI signals represent sound location in macaque auditory cortex. Neuron 93:971–983.e4. 10.1016/j.neuron.2017.01.013 28190642 PMC5757378

[B30] Parras GG, Nieto-Diego J, Carbajal GV, Valdés-Baizabal C, Escera C, Malmierca MS (2017) Neurons along the auditory pathway exhibit a hierarchical organization of prediction error. Nat Commun 8:2148. 10.1038/s41467-017-02038-6 29247159 PMC5732270

[B31] Pelli DG (1997) The VideoToolbox software for visual psychophysics: transforming numbers into movies. Spat Vis 10:437–442. 10.1163/156856897X00366 9176953

[B32] Rao RP (2005) Bayesian inference and attentional modulation in the visual cortex. Neuroreport 16:1843–1848. 10.1097/01.wnr.0000183900.92901.fc 16237339

[B33] Reynolds JH, Heeger DJ (2009) The normalization model of attention. Neuron 61:168–185. 10.1016/j.neuron.2009.01.002 19186161 PMC2752446

[B34] Schultz J, Pilz KS (2009) Natural facial motion enhances cortical responses to faces. Exp Brain Res 194:465–475. 10.1007/s00221-009-1721-9 19205678 PMC2755747

[B35] Spratling MW (2008) Predictive coding as a model of biased competition in visual attention. Vision Res 48:1391–1408. 10.1016/j.visres.2008.03.009 18442841

[B36] Summerfield C, de Lange FP (2014) Expectation in perceptual decision making: neural and computational mechanisms. Nat Rev Neurosci 15:745–756. 10.1038/nrn3838 25315388

[B37] Summerfield C, Egner T (2009) Expectation (and attention) in visual cognition. Trends Cogn Sci 13:403–409. 10.1016/j.tics.2009.06.003 19716752

[B38] Summerfield C, Trittschuh EH, Monti JM, Mesulam MM, Egner T (2008) Neural repetition suppression reflects fulfilled perceptual expectations. Nat Neurosci 11:1004–1006. 10.1038/nn.2163 19160497 PMC2747248

[B39] Vinken K, Op de Beeck HP, Vogels R (2018) Face repetition probability does not affect repetition suppression in macaque inferotemporal cortex. J Neurosci 38:7492–7504. 10.1523/JNEUROSCI.0462-18.2018 30030399 PMC6596142

[B40] Weiner KS, Sayres R, Vinberg J, Grill-Spector K (2010) fMRI-adaptation and category selectivity in human ventral temporal cortex: regional differences across time scales. J Neurophysiol 103:3349–3365. 10.1152/jn.01108.2009 20375251 PMC2888251

[B41] Willenbockel V, Sadr J, Fiset D, Horne GO, Gosselin F, Tanaka JW (2010) Controlling low-level image properties: the SHINE toolbox. Behav Res Methods 42:671–684. 10.3758/BRM.42.3.671 20805589

[B42] Zatorre RJ, Bouffard M, Ahad P, Belin P (2002) Where is 'where' in the human auditory cortex? Nat Neurosci 5:905–909. 10.1038/nn904 12195426

